# Venereal Excess, with Mental Impairment

**Published:** 1870-01

**Authors:** James S. Whitmire

**Affiliations:** Metamora, Illinois


ARTICLE IV. — *Venereal Excess, with Mental Impairment.* By JAMES S. WHITMIRE, Metamora, Illinois.

THERE have been, during the last few years, under my notice, several cases of diseases of the genital organs produced from excess, or rather their abuse; three of which presented different degrees of mental manifestations of derangement, all originating, probably, from the same cause, and resulting in physical and mental depravity.

The first, J. P., aged 26, farmer, married six years, and the father of two children. He consulted me during the month of March, 1867. He had a wild look out of his eyes, which were unsteady and vacant, and had a firm conviction that he had disease of the heart, which, he said, pained him, and insisted that it was swelled, and he maintained, persistently, the idea of impending dissolution. He was afraid to sleep; there was great restlessness and insomnia, so that his family and friends had to watch with him all night, and they informed me that they had not known him to sleep more than one hour at a time for more than a fortnight; his memory was accurately retentive of circumstances and dates, but he seemed to have no reasoning capabilties, and to have lost all filial and paternal impulses. His marriage relations had ever been of the most pleasant character; and this, probably, in connection with his lack of general intelligence, is what led him to excessive venereal indulgence, resulting in impotence, a shattered constitution and a disordered mind. He told me that he had not had a complete erection for more than six months, and consequently every attempt at copulation had proven unsuccessful.

Ever since this debility or laxity of his genital organs had existed, he had been troubled more or less with lecherous dreams during sleep, and consequently with nocturnal emissions, without the least sign or attempt at erection. This continuous drain on the nervous system, persisting after the impossibility of coition, still tended to render more permanent his mental and physical disabilities. His appetite had become impaired, his bowels obstinately constipated, and blood impoverished, so that, for all intents and purposes, he had become a mere vitalized wreck of frail humanity—a confirmed hypochondriac. His parents had procured the services of an irregular practitioner in the neighborhood, who had been tinkering with him for several months, and more than likely without the least conception of the real cause of the difficulty. What the diagnosis of the doctor was, or what his treatment, I am unable to say, but certain it was that the patient was growing more and more demented from day to day. Believing that all this train of evils had come from excessive venereal indulgence, and the nocturnal emissions from irritation, or rather, a peculiar irritability of the urethral canal about the mouths of the seminal ducts and the bulbous portion, I at once set to work to remedy the evil, by local as well as general treatment.

This condition of things is not unfrequently brought about in young men, from the vicious habit of masturbation, but, in twenty-four years of experience as a practitioner of medicine, I never before witnessed such a case in the marital state. In fact, I believe it is usually the case, that young men addicted to the habit, are recommended to marry, in order to avoid the evils consequent upon the practice. The indications for treatment in this case were clear enough, provided my theory of the difficulty was correct. It was, to lessen the nervous irritability of the bulbous and prostatic portion of the urethral canal; to enrich the blood; increase the powers of digestion, and to give general tone to the flagging energies of the system. My first step was, therefore, to thoroughly evacuate the bowels with a saline cathartic (they had been in a constipated condition for months, and never moved without the aid of medicines); next, I cauterized, with nitrate of silver the prostatic and bulbous portion of the urethral canal, by the use of Lallemand's porte caustique, and administered mucilaginous and demulcent drinks to obviate any inflammation that might arise from the use of the caustic. I regulated the bowels by a tonico-laxative pill given every night at bed-time, with the assistance of a cold-water enema in the morning, which was, at least, an adjuvant in restoring the tone of the debilitated organs. I gave tone to the nervous system, by the use of a ferruginous preparation, called the liquid oxy-sulph. of iron, the formula for which I found in the CHICAGO MEDICAL JOURNAL:

℞, Ferri Sulph, - - - - - - ʒ ij;

Acid Nit., - - - - - - - ʒ ij.

Rub well together, then add

Aqua, - - - - - - - ℥ iss.

In ℥ j of this solution, I dissolved quinine ʒ j, strychnia, grs. iij. Dose, six drops, in water, three times per day, before meals. The iron in this prescription served to enrich the blood, by the increase of the red corpuscles, the nitric acid and quinine to give tone to the digestive apparatus and increase assimilation, and the strychnia to give a fillip to the general nervous system by its specific action on the great centres, and, through them to increase the muscular tonicity of the bowels, which had become almost entirely paralyzed; and last, but not least, I administered bromide of potassium, grs. xx., in water, one hour after each meal, which tended to relieve the insomnia, produce sleep, and soothe the nervous irritability of the genital organs. I ordered beef tea alone, until such times as his appetite began to return and relish solid food, after which, he was allowed beef-steak, oysters, fruit, and roast potatoes. The foregoing treatment was steadily pursued for about three months, with the interruption of the bromide occasionally after meals, giving it, at such times, always at bedtime. At the end of two weeks, he began to show decided signs of improvement in every particular, and at the end of three months, to all appearances, he was perfectly restored to his family, with his usual mental equilibrium, and the proper vigor of all his other functions. He is now a successful grocer, doing business in one of our adjacent villages.

Another case, son of J. B., aged 19, farmer, had been complaining, and the family thought, acting curiously for six months. He finally lost his appetite, became dyspeptic, emaciated, taciturn, and had an idea that he was ill unto death, and must die; but did not know why, or what it was that was going to end his existence. The family had noticed that his mind was out of balance for some time, but there was no reason of which they knew that could have produced this condition, as he had neither been sick, nor received any injury.

As in the former case, the family had consulted an irregular practitioner regarding his condition, and he had been under his care for considerable time. Finding, however, that the young man's mind was growing gradually worse, I was consulted in regard to the case during the month of March, 1869. The family could give me no clue whatever to the difficulty, and the boy had no idea that his infirmities were brought on by his vicious habits. I accused him of such practices, but he persistently denied it. I was satisfied, however, from his general appearance, of what was the cause of his mental aberration, and accordingly told his father, who, of course, was taken quite aback, and had never suspected his son of such vicious practices. I directed that the young man should never be permitted to be out of the company of some of the family, and that his father should sleep with him every night. I cauterized the urethra, as in the former case, and put him under the same treatment, with the exception of the strychnine. In about four months, the boy was completely recovered, and is now a fat, hearty, cheerful young man, doing his accustomed labor on the farm.

Another case, William S., aged 22, a farmer, was a young man of good intelligence, and knew "what was the matter," and the cause of it. He had lost all interest in his business, and felt disinclined to any exertion whatever. He had lost his usual animation — so much so as to create much anxiety in the minds of his parents and friends. He had had nocturnal emissions for several months, so as to greatly annoy him, and had not had an erection for more than a year. He felt a natural delicacy in declaring his shame to his home physicians, and therefore resorted to some of the advertising charlatans of the city of Chicago for advice. After having made one or two trips to that city, and having been fleeced out of one or two hundred dollars by that delectable class of mountebanks, he concluded, on "sober, second thought," to try what virtue there was in home institutions. He therefore came to me, about two years ago, and laid his case before me. I put him through a very similar course to that of the former two cases, and in a very few months he had recovered his wonted ruddy complexion; his vigor and animal spirits had returned, and he was lively, energetic and active as ever. After his discharge, I never heard any complaints from him till this summer — 1869 — when he came to me, and, with considerable anxiety, said that he was entirely well, with one exception, and had been ever since his treatment two years previously. The exception was, that along with his spirits and wonted energy, he had never recovered the power of erection, remarking that he had never had a presentable penis for two years, and asked my advice as to the means of remedying the evil. I prescribed ten drops of diluted phosphoric acid, in water, three times per day, for one week, when it was to be increased one drop at each dose per day, till the maximum dose would be 25 drops, after which he was to see me again. At the end of this time, finding but little if any aphrodisiac powers in the acid, I prescribed acid phos. dil., hashish fluid ext., aa ʒ iv; of this the dose was twenty drops, three times per day, and increased one drop per day at each dose, till he took 40 gtt. At the same time that he began this prescription, I gave him the $\frac{1}{20}$ of a grain of strychnine, three times per day, and ordered the use of strychnine ointment to the perineum. About the time that he should have been taking 40 drops of the last prescription, I saw a notice of his marriage to an estimable young lady, which to me was conclusive evidence of his complete recovery. A few days ago I met him on the street, and made inquiries as to his welfare. "ALL RIGHT," said he; "I have been married now two months, and have no difficulty in performing all the duties of the marital relation." Of course, I advised prudence.

